# Uroporphyrinogen Decarboxylase as a Potential Target for Specific Components of Traditional Chinese Medicine: A Virtual Screening and Molecular Dynamics Study

**DOI:** 10.1371/journal.pone.0050087

**Published:** 2012-11-29

**Authors:** Yung-An Tsou, Kuan-Chung Chen, Hung-Che Lin, Su-Sen Chang, Calvin Yu-Chian Chen

**Affiliations:** 1 Department of Otolaryngology, China Medical University Hospital, Taichung, Taiwan; 2 School of Medicine, College of Medicine, China Medical University, Taichung, Taiwan; 3 Graduate Institute of Pharmaceutical Chemistry, China Medical University, Taichung, Taiwan; 4 School of Pharmacy, China Medical University, Taichung, Taiwan; 5 Department of Medical Research, China Medical University Hospital, Taichung, Taiwan; 6 Laboratory of Computational and Systems Biology, China Medical University, Taichung, Taiwan; 7 Department of Biotechnology, Asia University, Taichung, Taiwan; 8 Department of Biomedical Informatics, Asia University, Taichung, Taiwan; 9 China Medical University Beigang Hospital, Yunlin, Taiwan; National Cancer Institute at Frederick, United States of America

## Abstract

Uroporphyrinogen decarboxylase (UROD) has been suggested as a protectant against radiation for head and neck cancer (HNC). In this study, we employed traditional Chinese medicine (TCM) compounds from TCM Database@Taiwan (http://tcm.cmu.edu.tw/) to screen for drug-like candidates with potential UROD inhibition characteristics using virtual screening techniques. Isopraeroside IV, scopolin, and nodakenin exhibited the highest Dock Scores, and were predicted to have good Absorption, Distribution, Metabolism, Excretion, and Toxicity (ADMET) properties. Two common moieties, 2H-chromen-2-one and glucoside, were observed among the top TCM candidates. Cross comparison of the docking poses indicated that candidates formed stable interactions with key binding and catalytic residues of UROD through these two moieties. The 2H-chromen-2-one moiety enabled pi-cation interactions with Arg37 and H-bonds with Tyr164. The glucoside moiety was involved in forming H-bonds with Arg37 and Asp86. From our computational results, we propose isopraeroside IV, scopolin, and nodakenin as ligands that might exhibit drug-like inhibitory effects on UROD. The glucoside and 2H-chromen-2-one moieties may potentially be used for designing inhibitors of UROD.

## Introduction

Head and neck cancer (HNC), one of the most common malignancies worldwide [1], [2], refers to cancer originating from the upper aerodigestive tract [3]. Uroporphyrinogen decarboxylase (UROD) has been implicated as a tumor-selective protectant for HNC against radiation [4]. Inactivation of UROD coupled with radiation promoted *in vitro* apoptosis and cell cycle arrest of HNC cells. In addition, *in vivo* suppression of the tumor-forming ability of HNC cells and delayed growth of formed tumor xenografts in mice were reported [5]. These findings suggest that UROD may be a potential drug target for controlling HNC.

UROD, which is encoded by a single gene localized to the pter-p21 region of human chromosome 1 [6], [7], converts uroporphyrinogen III to coproporphyrinogen III through decarboxylation [8]–[12]. The catalytic process of decarboxylation starts with the acetate on the asymmetric ring of the natural substrate, uroporphyrinogen III, under physiological substrate concentrations [13], [14]. UROD is essential for biosynthesis of heme and chlorophyll [15]–[18], and exists as a stable homodimer in humans [19], [20]. Residues Arg37, Arg41 and His339 have been implied as key substrate binding residues, and Asp86, Tyr164 and Ser219 may be involved in binding or catalysis based on the crystal structure [21].

Traditional Chinese medicine (TCM) has been noted for its therapeutic usage in many diseases and novel candidate leads have been identified for anti-tumor, anti-viral, and stroke prevention among other therapeutic applications [22]–[24]. To identify potential UROD inhibitors from TCM, natural compounds in TCM Database@Taiwan (http://tcm.cmu.edu.tw/) [25] were employed for virtual screening. Each resulting candidate from molecular docking was tested for its absorption, distribution, metabolism, and excretion, toxicity (ADMET) properties. Molecular dynamics (MD) simulations were employed to examine the stabilizing interactions within each complex under a dynamic state simulating physiological conditions.

## Results and Discussion

### Docking

**Figure 1:**
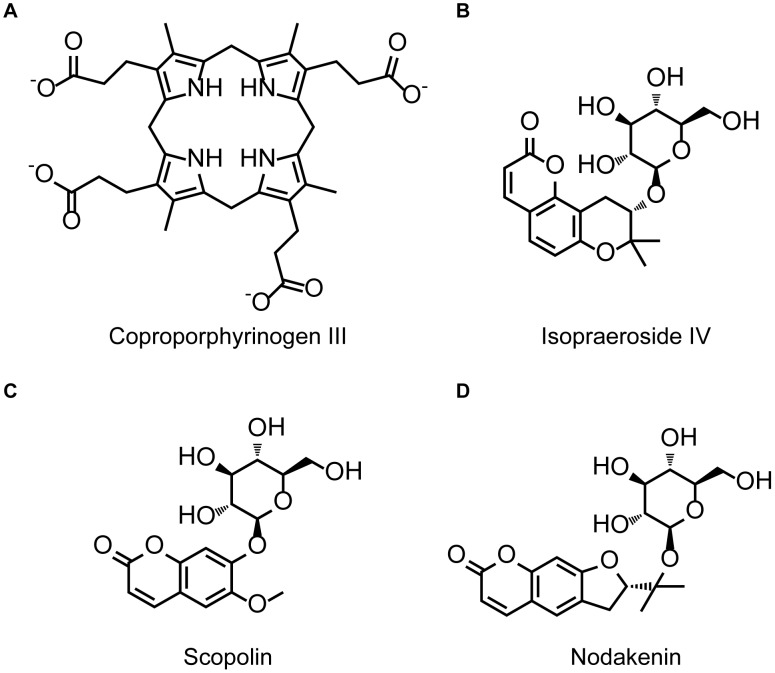
Scaffold of the control compound and TCM candidates. (A) Coproporphyrinogen III (B) isopraeroside IV (C) scopolin (D) nodakenin.

Dock Scores of the top seven TCM compounds and the control, coproporphyrinogen III, are listed in Table 1. The top TCM compounds were ranked according to Dock Score and all were calculated to have higher Dock Scores than coproporphyrinogen III. The top three TCM compounds isopraeroside IV, scopolin, and nodakenin were selected as candidates for further evaluation, and their respective scaffolds along with that of Coproporphyrinogen III are illustrated in Figure 1. Structural comparisons reveal that the TCM candidates share two common moieties, 2H-chromen-2-one and glucoside.

**Table 1:** Docking results of top TCM compounds and Coproporphyrin III.

Name	Dock Score
Isopraeroside IV	104.348
Scopolin	96.525
Nodakenin	95.998
Aurantiamide	95.191
9-hydroxy-(10E)-octadecenoic acid	95.088
8-hydroxy-(9E)-octadecenoic acid	93.675
Beauveriolide I	92.215
*Coproporphyrinogen III	91.919

* Control.

**Figure 2:**
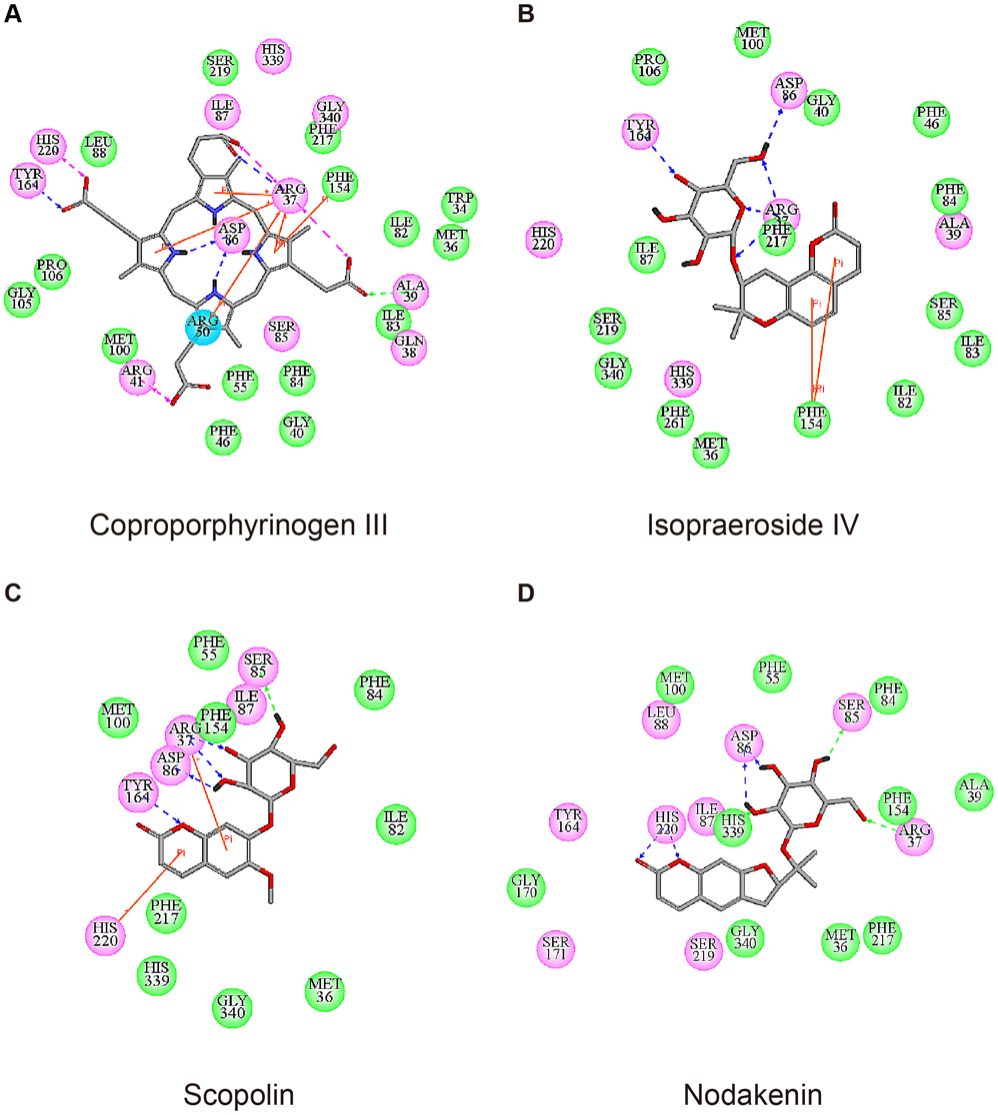
Docking pose of test ligands in UROD. (A) Coproporphyrinogen III (B) isopraeroside IV (C) scopolin (D) nodakenin. Orange solid lines and pink dashed lines represent pi interactions and charge interactions, respectively. H-bonds with amino acid main chains are shown in green and those with side chains are illustrated in blue. Magenta circles represent the residues involved in H-bond, charge, or polar interactions, and green circles represent residues involved in van der Waals interactions.

**Figure 3:**
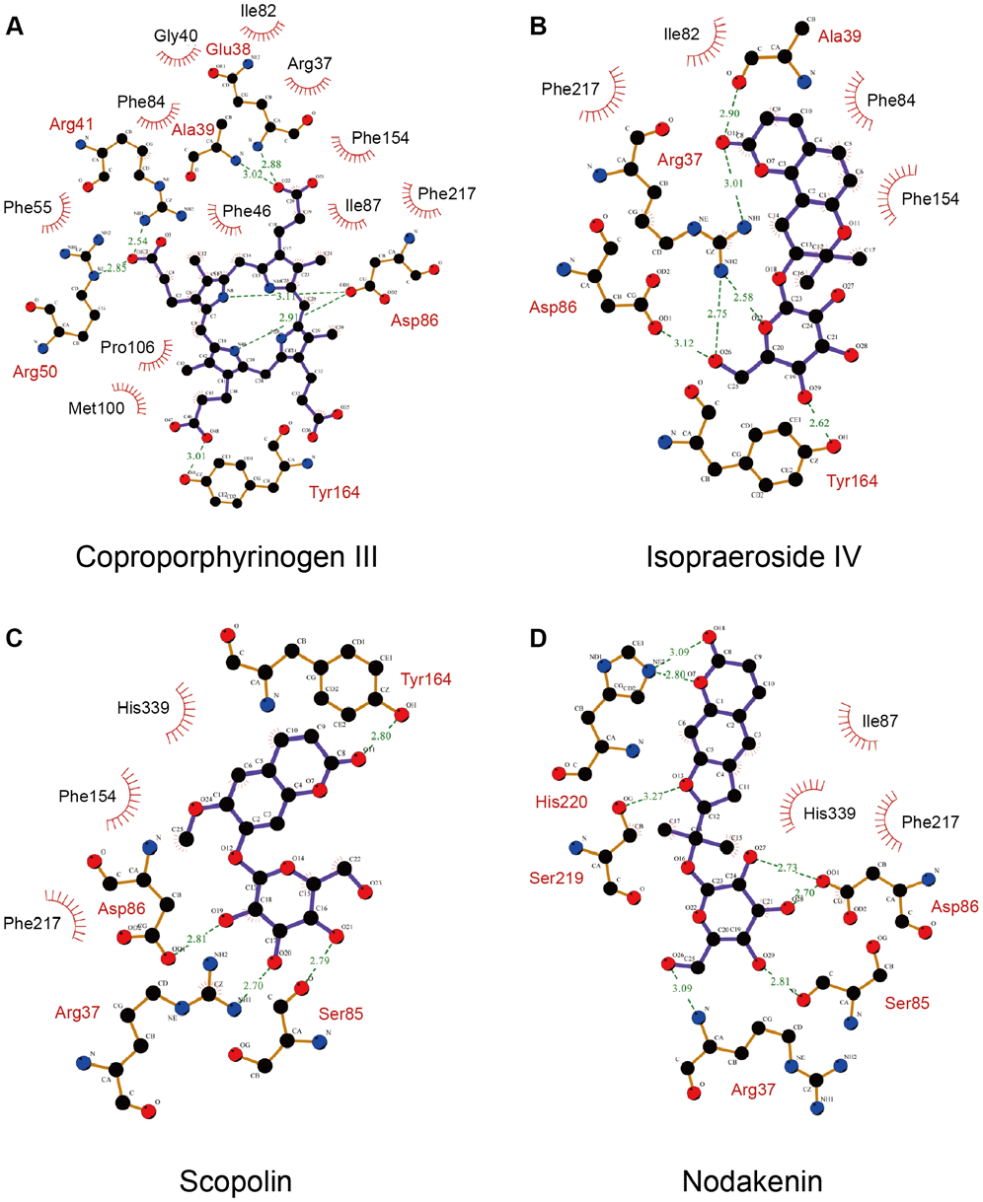
Interactions between test ligands and UROD binding site determined by LigPlot. (A) Coproporphyrinogen III (B) isopraeroside IV (C) scopolin (D) nodakenin.

Based on Swiss-Prot database, key binding and catalytic residues of uroporphyrinogen III in UROD include Phe55, Ser85, Asp86, Tyr164, Ser219, His339, and the region from Arg37 to Arg41 (UniProtKB: P06132) These are important residues with which the binding of our test ligands are compared against. Coproporphyrinogen III is the decarboxylated product of uroporphyrinogen III [8]–[12]. For clarification purposes, all interactions discussed within this study were based on computer simulation results. The decarboxylation of four acetate groups from uroporphyrinogen III reduced four moieties available for binding, therefore no interaction with Phe55, Ser85, Ser219, and His339 was observed (Figure 2A). Coproporphyrinogen III interacted with UROD binding site through pi-cation interactions with Arg37 and Arg50, pi-pi interaction with Phe154, and H-bonds with Arg37, Ala39, Asp86, and Tyr164. Ten amino acid residues were also involved in maintaining stability of coproporphyrinogen III within UROD via hydrophobic interactions (Figure 3A). For isopraeroside IV, pi-pi interactions with Phe154 in the 2H-chromen-2-one moiety and H-bonds with key residues, Arg37, Asp86, and Tyr164, in the glucoside moiety were detected (Figure 2B). Ligplot analysis further revealed H-bond formation of Arg37 and Ala39 with the 2H-chromen-2-one moiety (Figure 3B). No key residues were involved in the formation of hydrophobic interactions. Scopolin formed pi-cation interactions with Arg37 and His220 in the 2H-chromen-2-one moiety, and H-bonds with Arg37, Ser85, Asp86 in the glucoside moiety and Tyr164 in the 2H-chromen-2-one moiety (Figure 2C). These results were further supported by Ligplot analysis (Figure 3C). For nodakenin, H-bonds with Arg37, Ser85, Asp86 in the glucoside moiety, and His220 in the 2H-chromen-2-one moiety were observed (Figure 2D, Figure 3D). Regardless of interaction type, the docking poses indicate that Arg37 and Asp86 were key residues for TCM candidates. The 2H-chromen-2-one moiety of TCM candidates enabled pi interactions with key residues Arg37, Phe154, or His220, and the glucoside moiety formed H-bonds with key residues Arg37 and Asp86. Hydrophobic interactions with neighboring amino acid residues did not play a prominent stabilizing role for TCM candidates compared to coproporphyrinogen III (Figure 3).

### ADMET Properties

Pharmacokinetics properties of the candidates and control were subjected to computational evaluation using the ADMET Descriptors protocol of Discovery Studio 2.5 (DS 2.5). Results are summarized in Table 2. The predictions suggest that TCM candidates may have good to moderate absorption and ≥90% binding with plasma protein. Computational results also indicate that the candidates might have desirable drug like qualities such as low probabilities of inhibiting CYP2D6 or causing dose-dependent liver injuries. Blood brain barrier level predictions ranged widely, suggesting that drug delivery routes may need to be customized accordingly.

**Table 2:** Pharmacokinetic properties of top TCM compounds.

Name	AbsorptionLevel^1^	BBBLevel^2^	PPB Level^3^	CYP2D6 Probability^4^	Hepatotoxicity Probability^5^
Isopraeroside IV	1	4	2	0.356	0.417
Scopolin	1	4	2	0.346	0.496
Nodakenin	1	4	2	0.356	0.397
Aurantiamide	0	2	2	0.475	0.496
9-hydroxy-(10E)-octadecenoic acid	0	1	1	0.386	0.231
8-hydroxy-(9E)-octadecenoic acid	0	1	1	0.386	0.152
Beauveriolide I	1	4	1	0.475	0.463
*Coproporphyrinogen III	3	4	0	0.217	0.768

* Control.

1 Absorption level: 0-good absorption (within 95% confidence ellipse); 1-moderate absorption (within 99% confidence ellipse); 2-low absorption (outside 99% confidence ellipse).

2 BBB (blood-brain barrier) penetration levels: 0-very high; 1-high; 2-medium; 3-low; 4-undefined (outside 99% confidence ellipse).

3 Plasma Protein Binding: 1-binding >90%; 2-binding >95%.

4 Probability to inhibit Cytochrome P450 2D6.

5 Unlikely to cause dose-dependent liver injuries if <0.5.

### Molecular Dynamics Simulation

**Figure 4:**
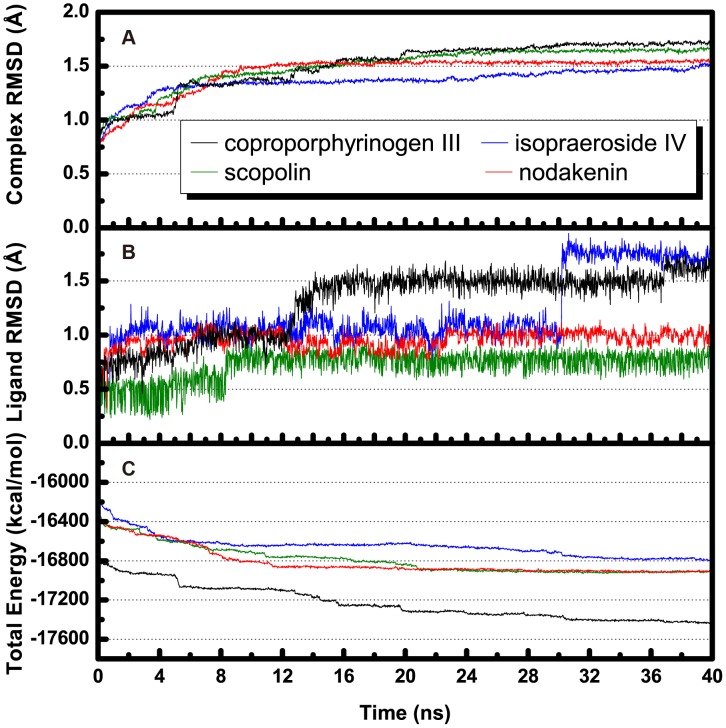
Trajectory changes during MD simulation. (A) Complex RMSD, (B) ligand RMSD, and (C) total complex energy.

**Figure 5:**
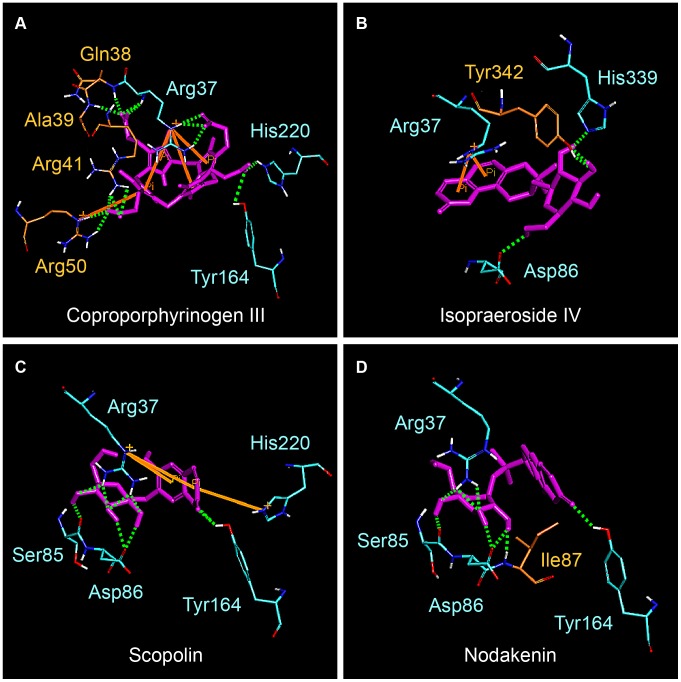
The average structure of docking poses with UROD during 38–40 ns MD. (A) Coproporphyrinogen III (B) isopraeroside IV (C) scopolin (D) nodakenin. Orange solid lines and green dashed lines represent pi-pi interactions and hydrogen bond interactions, respectively. Only polar hydrogens were shown within the illustrations for clarity.

Molecular dynamics (MD) simulation was conducted to evaluate stability of UROD-ligand complexes under dynamic conditions. Complex and ligand RMSD trajectories, which reflect atomic fluctuations, and total energy profiles of each complex are shown in Figure 4. Trajectories of protein-ligand complexes reached equilibrium after 37 ns, indicating complex stabilization after 37 ns. Figure 5 shows the average structures of each complex from 38–40 ns. Compared with its initial docking pose (Figure 2), coproporphyrinogen III formed H-bonds with Arg37, Gln38, Ala39, and Arg41 during MD. Pi-cation interactions with residues Arg37, Arg50, and His220 were stable for coproporphyrinogen III. Isopraeroside IV formed new pi-cation interactions with Arg37 during MD simulation. Pi-pi interactions with Phe154 by coproporphyrinogen III and isopraeroside IV were unstable and vanished during MD simulation. The 2H-chromen-2-one moiety of each candidate formed pi-cation interactions with Arg37.

**Figure 6:**
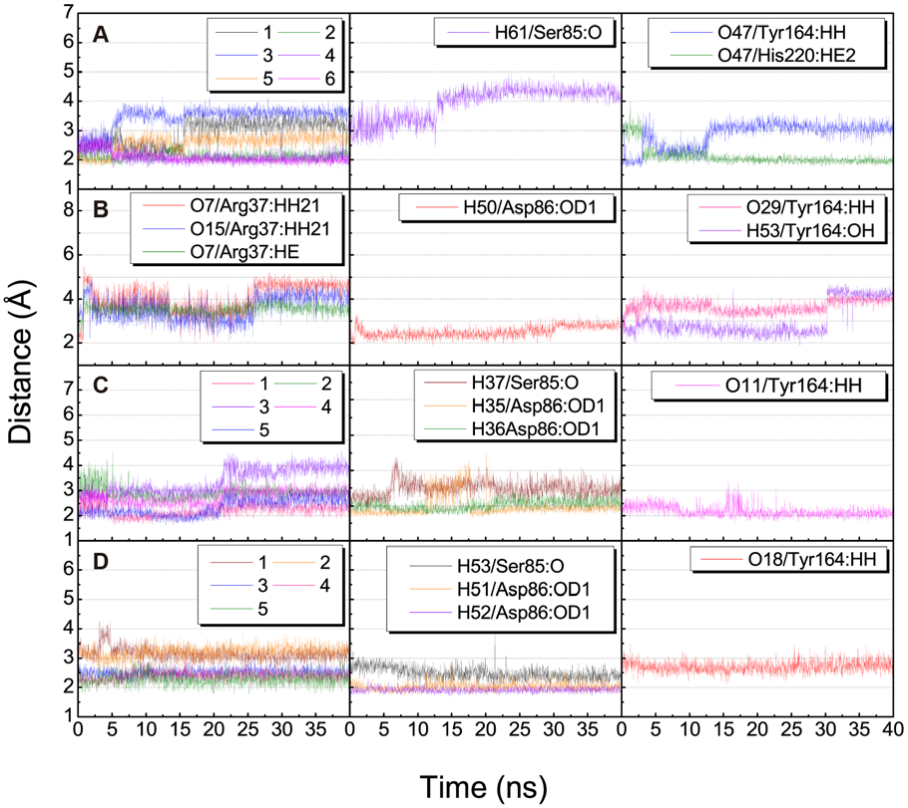
Distance (Å) of hydrogen bonds between UROD and test compounds. (A) Coproporphyrinogen III (B) isopraeroside IV (C) scopolin (D) nodakenin. Numbers in the legend refer to H-bond numberings of each respective ligand in Table 3.

H-bond variations of the TCM candidates with key residues Arg37, Ser85, Asp86, and Tyr164 are summarized in Table 3 and illustrated in Figure 6. Observations further suggested the importance of the glucoside moiety for stable binding. The glucoside moiety enabled stable H-bond formation with Asp86 in all candidates, and H-bonds with Arg37 in scopolin and nodakenin. For coproporphyrinogen III, the H-bond with Ser85 was not observed after 13 ns of MD. The H-bond between Tyr164 and isopraeroside IV was also lost after 30 ns of MD. This loss of H-bond corresponded to the sharp increase in ligand RMSD observed in Figure 4B. For nodakenin, the original H-bond with His220 was replaced by a stable H-bond with Tyr164 during MD. In summary, docking poses of the complexes after MD suggest that residue Arg37 is important for stabilizing the compounds within the binding site. The glucoside moiety of each candidate formed H-bonds with Arg37 and Asp86, and the 2H-chromen-2-one moiety of all but isopraeroside IV enabled H-bond formation with Tyr164.

**Table 3:** H-bond interactions of UROD with top TCM candidates and Coproporphyrinogen III.

Ligand	H-bond	Ligand Atom	Amino acid	Distance (Å)			H-bond occupancy
				Max.	Min.	Average	
Coproporphyrin III	1	O35	Arg37:HE	3.80	1.98	2.91	26.55%
	2	O36	Arg37:HE	2.79	1.75	2.12	98.65%
	3	O35	Arg37:HH21	4.13	1.94	3.45	3.30%
	4	O36	Arg37:HH21	3.13	1.71	2.09	94.40%
	5	O21	Arg37:HN	3.40	1.76	2.57	31.45%
	6	O22	Arg37:HN	2.95	1.72	2.08	94.05%
	7	H61	Ser85:O	4.95	2.44	3.95	0.30%
	8	O47	Tyr164:HH	3.63	1.73	2.86	24.30%
	9	O47	His220:HE2	4.03	1.77	2.11	91.30%
Isopraeroside IV	1	O7	Arg37:HH21	5.51	1.78	4.00	2.35%
	2	O15	Arg37:HH21	4.98	2.08	3.57	1.30%
	3	O7	Arg37:HE	4.51	2.43	3.53	0.10%
	4	H50	Asp86:OD1	3.34	1.84	2.52	50.95%
	5	O29	Tyr164:HH	4.58	2.15	3.65	0.20%
	6	H53	Tyr164:OH	4.71	1.89	3.02	27.30%
Scopolin	1	O20	Arg37:HH12	3.21	1.63	2.22	85.55%
	2	O21	Arg37:HH12	4.54	2.29	2.91	1.50%
	3	O19	Arg37:HH21	4.58	2.39	3.38	0.45%
	4	O19	Arg37:HH22	3.58	2.08	2.75	19.90%
	5	O20	Arg37:HH22	3.31	1.63	2.33	66.15%
	6	H37	Ser85:O	3.41	1.83	2.47	56.80%
	7	H35	Asp86:OD1	3.47	1.67	1.99	89.30%
	8	H36	Asp86:OD1	2.76	1.69	2.02	99.75%
	9	O11	Tyr164:HH	3.37	1.73	2.18	90.95%
Nodakenin	1	O26	Arg37:HH12	4.24	2.43	3.15	0.10%
	2	O29	Arg37:HH12	3.93	2.48	3.20	0.05%
	3	O28	Arg37:HH21	2.97	1.98	2.51	46.35%
	4	O28	Arg37:HH22	3.02	1.86	2.40	74.15%
	5	O29	Arg37:HH22	3.10	1.77	2.27	85.50%
	6	H53	Ser85:O	3.88	1.92	2.48	56.85%
	7	H51	Asp86:OD1	2.74	1.73	2.02	99.30%
	8	H52	Asp86:OD1	2.31	1.71	1.92	100.00%
	9	O18	Tyr164:HH	3.47	2.12	2.71	13.15%

**H-bond occupancy cutoff: 2.5 Å.**

**Figure 7:**
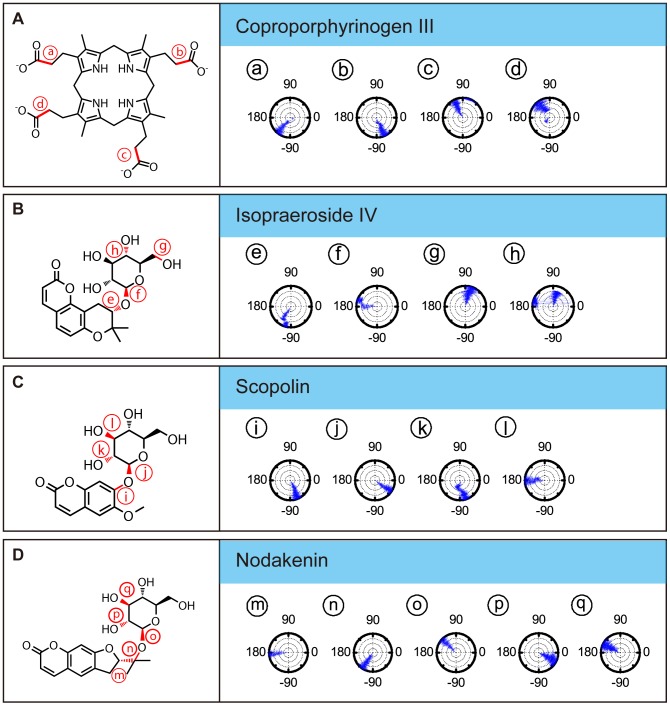
Torsion angles of test ligands in UROD complex. (A) Coproporphyrinogen III (B) isopraeroside IV (C) scopolin (D) nodakenin.

The importance of Arg37 and Asp86 for TCM candidate binding were further supported by torsion analysis results. The torsion shown for coproporphyrinogen III at **a** and **d** represent carboxyl groups that form H-bonds with Arg37. Location **d** is clearly more unstable (Figure 7A). Isopraeroside IV, scopolin, and nodakenin form H-bonds with Arg37 through the glycoside moiety. Torsion of isopraeroside IV indicate little fluctuation at **e** and **f** during MD, torsion at **g** shows that isopraeroside IV can form continuous H-bonds with Arg37 (Figure 7B). Torsions measured at **i** and **j** for scopolin indicated that there was little fluctuation, and torsions at **l** indicate that its hydroxyl group and Arg37 are capable of forming H-bonds during MD (Figure 7C). Scopolin also formed pi-cation interactions with Arg37 (Figure 2C), providing support that scopolin can form stable interactions with this amino acid. Nodakenin interacts with Arg37 through its glycoside moiety. As shown by the torsions at **m**, **n**, and **o**, the glycosidic moiety of nodakenin remained stable (Figure 7D). Results of torsion analysis show that the TCM candidates form stable bonds with Arg37, the primary binding residue of coproporphyrinogen III.

Asp86 is another key residue within the UROD binding site with which the secondary amine group of coproporphyrinogen III forms H-bonds (Figure 3A). Torsion changes observed at **c** within the final 40 ns indicated large rotations, implying unstable bond formation. Similarly, TCM candidates also formed H-bonds with Asp86. Torsion **g** refers to rotational changes measured for the hydroxyl group of isopraeroside IV which forms an H-bond with Asp86 (Figure 7B). As indicated in Figure 3B, this hydroxyl group also interacted with Arg37. Torsion **g** was stable throughout the MD simulation, indicating that stable bonds were formed with Asp86 in addition to Arg37. Torsion **k** measures the H-bond changes formed between Asp86 and scopolin (Figure 3C, Figure 7C). Consistency of the torsion from 20 ns to 40 ns supports the ability of the hydroxyl group of scopolin to form stable interactions with Asp86. H-bonds were detected between nodakenin and Asp86 (Figure 3D). Torsions at **p** and **q** show rotation of the two hydroxyl groups on nodakenin which bond with Asp86. No obvious changes were observed during the 40 ns MD.

Residues Tyr164, Ala39, Phe154, and His220 also seem to play important roles for maintaining TCM candidates within the UROD binding site. Coproporphyrinogen III has limited fluctuations at **b** and **d** (Figure 7A), suggesting that the carboxyl groups with Tyr164 and Ala39 can form stable H-bonds. Isopraeroside IV formed H-bonds with Tyr164 and Ala39, but torsion at **h** showed that the carbonyl group could not maintain a stable H-bond with Tyr164 (Figure 7B). Distance trajectories also show that the H-bond with Tyr164 averaged around 4 Å (Figure 6B), leaving only hydrophobic interactions to stabilize the 2H-chromen-2-one moiety of isopraeroside IV. By contrast, the H-bonds formed between scopolin and Tyr164 range within 2–3 Å (Figure 6C), suggesting a more stable interaction. With regard to Tyr164, affinity of scopolin is higher than that of isopraeroside IV. However, isopraeroside IV can form interactions with Ala39 (Figure 7B) which were not observed in scopolin (Figure 7C). Phe154 interacted with coproporphyrinogen III (Figure 2A) and isopraeroside IV (Figure 2B) in the form of pi-pi interactions. Ligplot analysis indicates that Phe154 formed hydrophobic interactions with isopraeroside IV (Figure 7B) and scopolin (Figure 7C). His220 is an H-bond forming residue for the carbonyl group of nodakenin (Figure 2D and Figure 7D). In summary, Arg37 and Asp86 are likely key residues for designing UROD inhibitors. Other amino acids Tyr164, Ala39, Phe154, His220 are residues that aid in forming stabilizing interactions, and should be taken into consideration to enable designed inhibitors to bind to the UROD binding site.

**Figure 8:**
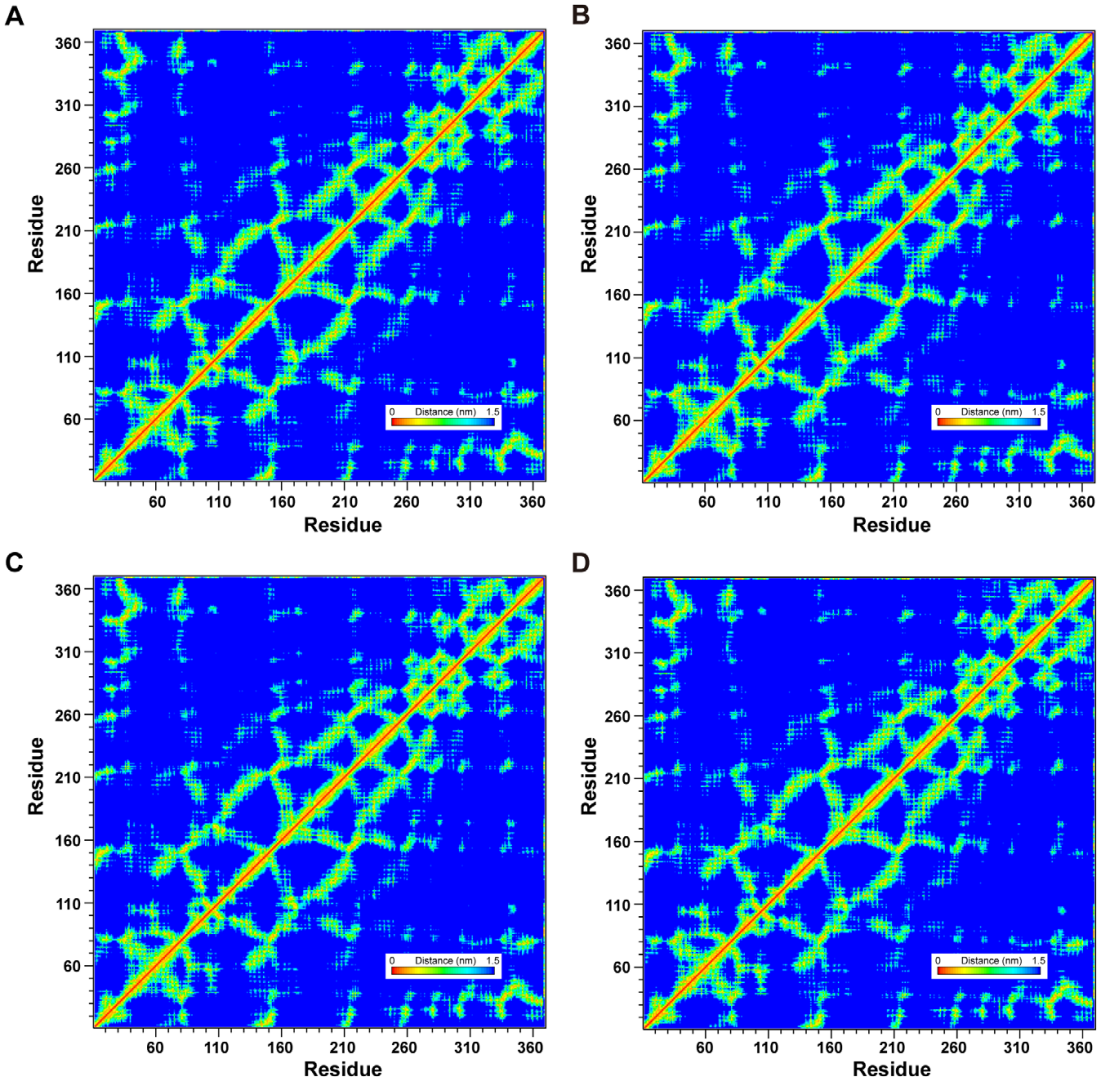
Matrices of average amino acid distance measured during 40 ns MD. Matrices given represent the average amino acid distances in protein-ligand complexes formed between UROD and (A) Coproporphyrinogen III (B) isopraeroside IV (C) scopolin (D) nodakenin. Distance matrices were generated by GROMACS.

**Figure 9:**
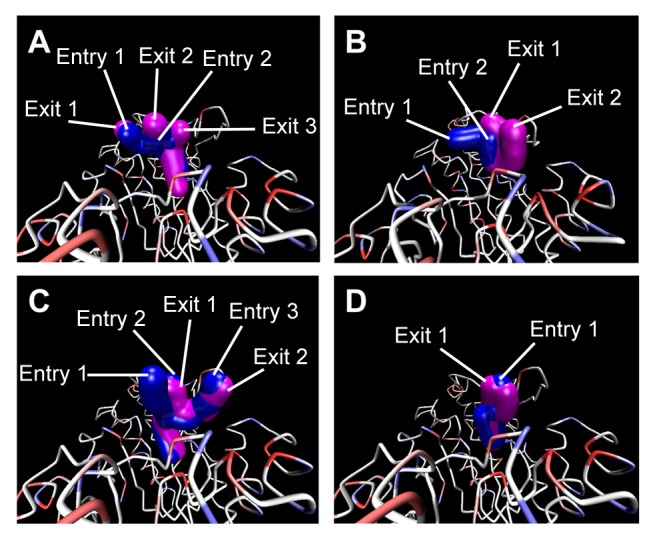
Snapshots of simulated passageways for selected ligands to the binding site in UROD. Passageways for (A) Coproporphyrinogen III (B) isopraeroside IV (C) scopolin (D) nodakenin are presented. Pathways calculated from MD confirmations during 0–5 ns are shown in blue and designated as entry pathways. Pathways calculated from MD confirmations during 35–40 ns are shown in magenta and designated as exit pathways. LigandPath was used to calculate potential pathways using a minimum clearance of 2.5 Å and a surface probe of 4 Å.

Global topology of UROD was not affected regardless of binding with coproporphyrinogen III (Figure 8A) or our proposed TCM candidates (Figure 8B, 8C, and 8D) since no significant differences were observed in the smallest distance matrices of the four complexes. LigandPath results (Figure 9) show that all test ligands were projected to have access to (“entry” passageways) and from (“exit” passageways) the designated binding site based on conformation ensembles formed by the initial and final 5 ns of MD simulation, respectively.

### Conclusion

The residues of Phe55, Ser85, Asp86, Tyr164, Ser219, His339, and the region from Arg37 to Arg41 are key binding and catalytic residues of UROD. Docking poses suggest that Arg37 was important to maintain ligand position within the binding site. TCM candidates isopraeroside IV and scopolin formed pi-cation interactions with Arg37 through the 2H-chromen-2-one moiety. Scopolin and nodakenin formed H-bonds with Arg37 and Asp86 in the glucoside moiety and Tyr164 via the 2H-chromen-2-one moiety. These interactions may potentially inhibit binding of the natural substrate, uroporphyrinogen III. From the results of this study, we propose TCM compounds, isopraeroside IV, scopolin, and nodakenin as drug-like compounds with potential as UROD inhibitors. The TCM candidates were predicted with good pharmacokinetic characteristics in addition to competitive binding characteristics. The glucoside and 2H-chromen-2-one moieties enhance ligand-UROD binding and are important moieties for potential inhibitors of UROD.

## Materials and Methods

### Data Collection

The crystal structure of human uroporphyrinogen decarboxylase (UROD) monomer (PDB ID: 1URO) [21] used in this study was obtained from Research Collaboratory for Structural Bioinformatics (RCSB) Protein Data Bank. A total of 9,029 molecules from TCM Database@Taiwan [25] which passed Lipinski's Rule of Five [26] were used for screening. Each compound was adjusted to its proper ionization state under physiological pH using Accelrys DS 2.5.

### Docking

Virtual docking simulation under Chemistry at HARvard Molecular Mechanics (CHARMm) force field [27] was performed by LigandFit module [28] of DS 2.5. The natural product of UROD, coproporphyrinogen III, was used as a control. Candidate ligands were chosen based on their Dock Score and evaluated for their pharmacokinetics properties. The Absorption, Distribution, Metabolism, Excretion, and Toxicity (ADMET) properties were evaluated by ADMET Descriptors protocol of DS 2.5. Interactions between each candidate ligand and UROD binding site were evaluated using LigPlot v.2.2.25 [29].

### Molecular Dynamics Simulation

Molecular dynamics (MD) simulation was performed using the Simulation package of DS 2.5 under CHARMm force field [27]. The time step for the entire MD simulation was set at 0.002 ps. The SHAKE algorithm was applied to constrain all bonds involving hydrogen atoms. Following minimization with Steepest Descent [30] and Conjugate Gradient [31] at maximum cycles of 6,000 each, the system was gradually heated from 50 K to 310 K within 50 ps and equilibrated for 200 ps. The NVT (canonical ensemble) with a Berendsen thermal coupling method temperature coupling decay time of 0.4 ps was performed for 40 ns. Analyze Trajectory module in DS 2.5 was used to analyze MD trajectories and applied to examine ligand/complex RMSDs, H-bond distances, MD dock poses, and torsion fluctuations. GROMACS was used to analyze secondary structure changes and calculate average amino acid distances recorded during the 40 ns MD. LigandPath, which is a simplified, user-interface version of Dynamic Map Ensemble (DyME) [32], was applied to identify possible ligand passageways through Voronoi diagram. For each MD conformation within the selected time frame, Voronoi diagram partitions the free space within the protein to have equal distance between each atom. Multiple MD conformations are then combined to form an ensemble which provides dynamic information on available passageways over a given period of time. For our purposes, the minimum clearance was set at 2.5 Å and the surface probe was 4 Å. Passageways calculated using time frames from 0–5 ns were designated as entries and those calculated from time frames from 35–40 ns were designated as exits.
